# Real-Time Tracking of Single and Multiple Objects from Depth-Colour Imagery Using 3D Signed Distance Functions

**DOI:** 10.1007/s11263-016-0978-2

**Published:** 2017-01-11

**Authors:** C. Y. Ren, V. A. Prisacariu, O. Kähler, I. D. Reid, D. W. Murray

**Affiliations:** 1 Department of Engineering Science, University of Oxford, Oxford, UK; 2 School of Computer Science, University of Adelaide, Adelaide, Australia

**Keywords:** Multi-object tracking, Depth tracking, RGB-D imagery, Signed distance functions, Real-time

## Abstract

We describe a novel probabilistic framework for real-time tracking of multiple objects from combined depth-colour imagery. Object shape is represented implicitly using 3D signed distance functions. Probabilistic generative models based on these functions are developed to account for the observed RGB-D imagery, and tracking is posed as a maximum a posteriori problem. We present first a method suited to tracking a single rigid 3D object, and then generalise this to multiple objects by combining distance functions into a shape union in the frame of the camera. This second model accounts for similarity and proximity between objects, and leads to robust real-time tracking without recourse to bolt-on or ad-hoc collision detection.

## Introduction

Tracking object pose in 3D is a core task in computer vision, and has been a focus of research for many years. For much of that time, model-based methods were concerned with rigid objects having simple geometrical descriptions in 3D and projecting to a set of sparse and equally simple features in 2D. The last few years have seen fundamental changes in every aspect, from the use of learnt, geometrically complex, and sometimes non-rigid objects, to the use of dense and rich representations computed from conventional image and depth cameras.

In this paper we focus on very fast tracking of multiple rigid objects, without placing arbitary constraints upon their geometry or appearance. We first present a revision of our earlier 3D object tracking method using RGB-D imagery (Ren et al. 2013). Like many current 3D trackers, this was developed for single object tracking only. An extension to multiple objects could be formulated by replicating multiple independent object trackers, but such a naïve approach would ignore two common pitfalls. The first is similarity in appearance: multiple objects frequently have similar colour and shape (hands come in pairs; cars are usually followed by more cars, not by elephants; and so on). The second is the hard physical constraint that multiple rigid bodies may touch but may not occupy the same 3D space. These two issues are addressed here in an RGB-D tracker that we originally proposed in Ren et al. (2014). This tracker can recover the 3D pose of multiple objects with *identical* appearance, while preventing them from intersecting. The present paper summarizes our previous work and places the single and multiple object trackers in a common framework. We also extend the discussion of related work, and present additional experimental evaluations.

The paper is structured as follows. Section 2 gives an overview of related work. Sections 3 and 4 detail the probabilistic formulation of the single object tracker and the extensions to the multiple object tracking problem. Section 5 discusses the implementation and performance of our method and Sect. 6 provides experimental insight into its operation. Conclusions are drawn in Sect. 7.

## Related Work

We begin our discussion by covering the general theme of model-based 3D tracking, then consider more specialised works that use distance transforms, and detail methods that aim to impose physical constraints for multi object tracking.

Most existing research on 3D tracking, with or without depth data, uses a model-based approach, estimating pose by minimising an objective function which captures the discrepancy between the expected and observed image cues. While limited computing power forced early authors (e.g. Harris and Stennett 1990; Gennery 1992; Lowe 1992) to exploit highly sparse data such as points and edges, the use of dense data is now routine.

An algorithm commonly deployed to align dense data is Iterative Closest Point (Besl and McKay 1992). ICP is used by Held et al. (2012) who input RGB-D imagery from a Kinect sensor to track hand-held rigid 3D puppets. They achieve robust and real-time performance, though occlusion introduced by the hand has to be carefully managed through a colour-based pre-segmentation phase. Rather awkwardly, a different appearance model is required to achieve this pre-segmentation when tracking multiple objects. A more general work is KinectFusion (Newcombe et al. 2011), where the entire scene structure along with camera poses are estimated simultaneously. Ray-casting is used to establish point correspondences, after which estimation of alignment or pose is achieved with ICP. However, a key requirement when tracking with KinectFusion is that the scene moves rigidly with respect to the camera, a condition which is obviously violated when generalising tracking to multiple independently moving objects.

**Fig. 1:**
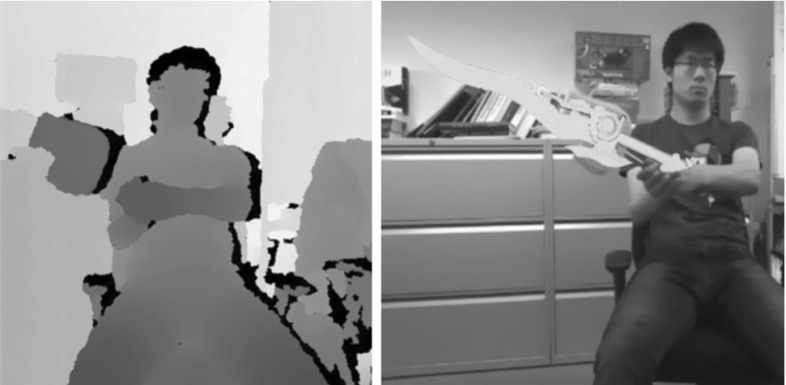
Illustration of our method tracking an arbitrary object and enabling its use as a game controller. On the *left* we show the depth image overlaid with the tracking result and on the *right* we visualise a virtual sword with the corresponding 3D pose overlaid on the RGB image

Kim et al. (2010) perform simultaneous camera and multi-object pose estimation in real-time using only colour imagery as input. First, all objects are placed statically in the scene, and a 3D point cloud recovered and camera pose initialized by triangulating matched SIFT features (Lowe 2004) in a monocular keyframe reconstruction (Klein and Murray 2007). Second, the user delineates each object by drawing a 3D box on a keyframe, and the object model is associated with the set of 3D points lying close to the surfaces of the 3D boxes. Then, at each frame, the features are used for object re-detection, and a pose estimator best fits the detected object’s model to the SIFT features. The bottom-up nature of the work rather limits overall robustness and extensibility. With the planar model representation used, only cuboid-shaped objects can be tracked.

A number of related tracking methods—and ones which appear much more readily generalisable to multiple objects—use sampling to optimise pose. In each the objective function involves rendering the model at some hypothesised pose into the observation domain and evaluating the differences between the generated and the observed visual cues; but in each the cost is deemed too non-convex, or its partial derivatives too expensive or awkward to compute, for gradient-based methods to succeed. Particle Swarm Optimization was used by Oikonomidis et al. (2011a) to track an articulated hand, and by Kyriazis and Argyros (2013) to follow the interaction between a hand and an object. Both achieve real-time performance by exploiting the power of GPUs, but the level of accuracy that can be achieved by PSO is not thoroughly understood either empirically or theoretically. Particle filtering has also been used, and with a variety visual features. Recalling much earlier methods, Azad et al. (2011) match 2D image edges with those rendered from the model, while Choi and Christensen (2010) add 2D landmark points to the edges. Turning to depth data, the objective function of Ueda (2012) compares the rendered and the observed depth map, while Wuthrich et al. (2013) also model the per-pixel occlusion and win more robust tracking in presence of occlusion. Adding RGB to depth, Choi and Christensen (2013) fold in photometric, 3D edge and 3D surface normal measures into their likelihood function for each particle state. Real-time performance is achieved using GPUs, but nonetheless careful limits have to be placed on the number of particles deployed.

**Fig. 2:**
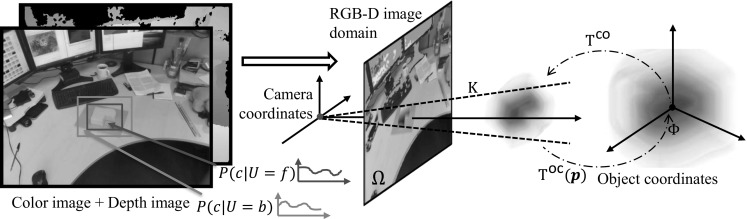
Representation of the 3D model $\varPhi$, the RGB-D image domain $\varOmega$, the foreground/background models $P(c|U{=}f)$, $P(c|U=b)$ and the pose $\texttt {T}^{\mathsf{co}}(\mathbf{p})$

An alternative to ICP is the use of the signed distance function (SDF). It was first shown by Fitzgibbon (2001) that distance transforms could be used to register 2D/3D point sets efficiently. Prisacariu and Reid (2012) project a 3D model into the image domain to generate an SDF-like embedding function, and the 3D pose of a rigid object is recovered by evolving this embedding function. A faster approach has been linked with a 3D reconstruction stage, both without depth data by Prisacariu et al. (2012, 2013) and with depth by Ren et al. (2013). The SDF was used by Ren and Reid (2012) to formulate different embedding functions for robust real-time 3D tracking of rigid objects using only depth data, an approach extended by Ren et al. (2013) to leverage RGB data in addition. A similar idea is described by Sturm et al. (2013), who use the gradient of the SDF directly to track camera pose. KinectFusion (Newcombe et al. 2011) and most of its variants use a truncated SDF for shape representation, but, as noted earlier, KinectFusion uses ICP for camera tracking rather than directly exploiting the SDF. As shown by Sturm et al. (2013), ICP is less effective for this task.

Physical constraints in 3D object tracking are usually enforced by reducing the number of degrees of freedom (dof) in the state. An elegant example of tracking of connected objects (or sub-parts) in this way is given by Drummond and Cipolla (2002). However, when tracking multiple independently moving objects, physical constraints are introduced suddenly and intermittently by the collision of objects, and cannot be conveniently enforced by dof reduction. Indeed, rather few works explicitly model the physical collision between objects. Oikonomidis (2012) tracks two interacting hands with Kinect input, introducing a penalty term measuring the inter-penetration of fingers to invalidate impossible articulated poses. Both Oikonomidis et al. (2011b) and Kyriazis and Argyros (2013) track a hand and moving object simultaneously, and invalid configurations similarly penalized. In both cases the measure used is the minimum magnitude of 3D translation required to eliminate intersection of the two objects, a measure computed using the Open Dynamic Engine library (Smith 2006). In contrast, in the method presented here the collision constraint is more naturally enforced through a probabilistic generative model, without the need of an additional physics simulation engine (Fig.1).

## Single Object Tracking

Sections 3.2 and 3.3 introduce the graphical model and develop the maximum a posterior estimation underpinning our 3D tracker; and in Sect. 3.4 we discuss the online learning of the appearance model. First though we describe the basic geometry of the scene and image, sketched in Fig. 2, and establish notation.

### Scene and Image Geometry

Using calibrated values of the intrinsic parameters of the depth and colour cameras, and of the extrinsics between them, the colour image is reprojected into the depth image. We denote the aligned RGB-D image as

1 $$\begin{aligned} \varOmega =\left\{ \{\mathbf {X}^{\mathsf{i}}_1, c_1\},\{\mathbf {X}^{\mathsf{i}}_2, c_2\}\dots \{\mathbf {X}^{\mathsf{i}}_{N_\varOmega }, c_{N_\varOmega }\}\right\} ~, \end{aligned}$$

where $\mathbf {X}^{\mathsf{i}}= Z\,\mathbf {x}= [Zu, Zv, Z]^\top$ is the homogeneous coordinate of a pixel with depth *Z* located at image coordinates [*u*, *v*], and *c* is its RGB value. (The superscripts $\mathsf{i}$, $\mathsf{c}$ and $\mathsf{o}$ will distinguish image, camera and object frame coordinates).

**Fig. 3:**
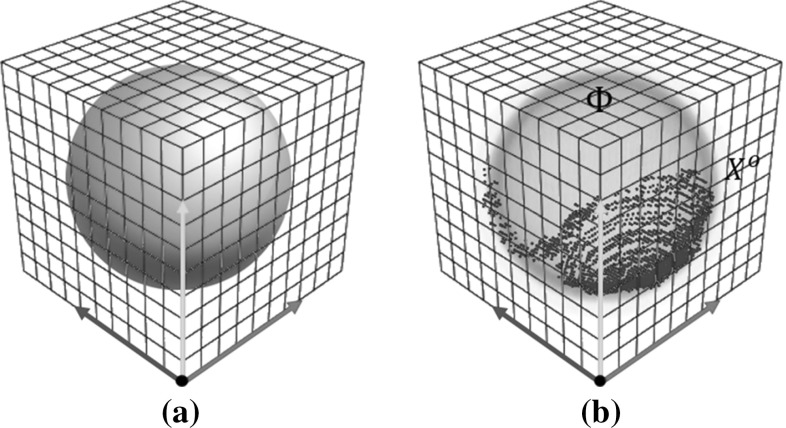
**a** An object defined in a voxelised space. **b** Its signed distance embedding function is also defined in object coordinates with the same voxelisation

As illustrated in Fig. 3, we represent an object model by a 3D signed distance function (SDF), $\varPhi$, in object space. The space is discretised into voxels on a local grid surrounding the object. Voxel locations with negative signed distance map to the inside of the object and positive values to the outside. The surface of the 3D shape is defined by the zero-crossing $\varPhi =0$ of the SDF.

A point $\mathbf {X}^{\mathsf{o}}=[X^\mathsf{o},Y^\mathsf{o},Z^\mathsf{o},1]^\top$ on an object with pose $\mathbf{p}$, composed of a rotation and translation $\{\mathtt{R},\mathbf {t}\}$, is transformed into the camera frame as $\mathbf {X}^{\mathsf{c}}= \texttt {T}^{\mathsf{co}}(\mathbf{p}) \mathbf {X}^{\mathsf{o}}$ by the $4\times 4$ Euclidean transformation $\texttt {T}^{\mathsf{co}}(\mathbf{p})$, and projected into the image under perspective as $\mathbf {X}^{\mathsf{i}}= \texttt {K} [\texttt {I}_{3\times 3}|\mathbf{0}] \mathbf {X}^{\mathsf{c}}$, where $\mathtt{K}$ is the depth camera’s matrix of intrinsic parameters.

We introduce a co-representation $\left\{ \mathbf {X}^{\mathsf{i}},c,U \right\}$ for each pixel, where the label $U\in \{f, b\}$ is set depending on whether the pixel is deemed to originate from the foreground object or from the background. Two appearance models describe the colour statistics of the scene: that for the foreground is generated by the object surface, while that for the background is generated by voxels outside the object. The models are represented by the likelihoods $P(c|U=f)$ and $P(c|U=b)$ which are stored as normalised RGB histograms using 16 bins per colour channel. The histograms can be initialised either from a detection module or from a user-selected bounding box on the RGB image, in which the foreground model is built from the interior of the bounding box and the background from the immediate region outside the bounding box.

**Fig. 4:**
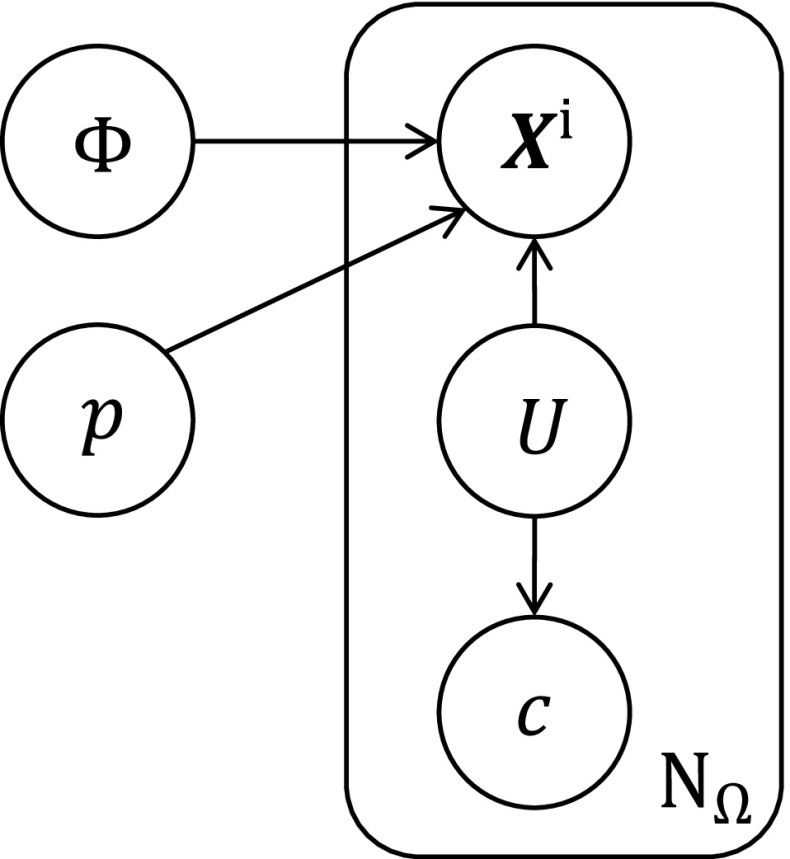
The graphical model underpinning the single-object tracker

### Generative Model and Tracking

The generative model motivating our approach is depicted in Fig. 4. We assume that each pixel is independent, and sample the observed RGB-D image $\varOmega$ as a bag-of-pixels $\{\mathbf {X}^{\mathsf{i}}_j,c_j\}_{1 \ldots N_{\varOmega }}$. Each pixel depends on the shape $\varPhi$ and pose $\mathbf{p}$ the object, and on the per-pixel latent variable $U_j$. Strictly, it is the depth $Z(\mathbf {x}_j)$ and colour $c_j$ that are randomly drawn for each pixel location $\mathbf {x}_j$, but we use $\mathbf {X}^{\mathsf{i}}_j$ as a convenient proxy for $Z(\mathbf {x}_j)$.

Omitting the index *j*, the joint distribution for a single pixel is

2 $$\begin{aligned}&P(\mathbf {X}^{\mathsf{i}},c,U,\varPhi ,\mathbf{p}) \nonumber \\&\quad = P(\varPhi )\,P(\mathbf{p})\, P(\mathbf {X}^{\mathsf{i}}|U,\varPhi ,\mathbf{p})\, P(c|U)\,P(U) \end{aligned}$$

and marginalising over the label *U* gives

3 $$\begin{aligned}&P(\mathbf {X}^{\mathsf{i}},c,\varPhi ,\mathbf{p})=P(\varPhi )P(\mathbf{p})\nonumber \\&\quad \sum _{u\in \{f,b\}} P(\mathbf {X}^{\mathsf{i}}|U=u,\varPhi ,\mathbf{p})P(c|U=u)P(U=u). \end{aligned}$$

**Fig. 5:**
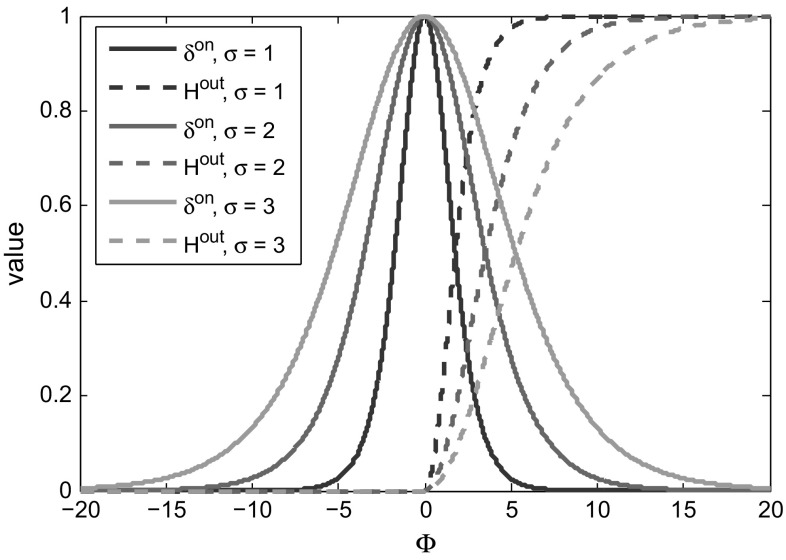
The smoothed delta $\delta ^{\mathsf{on}}$ and Heaviside $H^{\mathsf{out}}$ functions

Given the pose, $\mathbf {X}^{\mathsf{o}}$ can be found immediately as the back- projection of $\mathbf {X}^{\mathsf{i}}$ into object coordinates

4 $$\begin{aligned} \mathbf {X}^{\mathsf{o}}= \texttt {T}^{\mathsf{oc}}(\mathbf{p})[ (\texttt {K}^{-1}\mathbf {X}^{\mathsf{i}})^\top \, {1}]^\top ~, \end{aligned}$$

so that $P(\mathbf {X}^{\mathsf{i}}|U=u,\varPhi ,\mathbf{p}) \equiv P(\mathbf {X}^{\mathsf{o}}|U=u,\varPhi ,\mathbf{p})$. This allows us to define the per-pixel likelihoods as functions of $\varPhi (\mathbf {X}^{\mathsf{o}})$: we use a normalised smoothed delta function and a smoothed, shifted Heaviside function

5 $$\begin{aligned} P(\mathbf {X}^{\mathsf{i}}|U{=}f,\varPhi ,\mathbf{p})&= \delta ^{\mathsf{on}}(\varPhi (\mathbf {X}^{\mathsf{o}}))/\eta _f\end{aligned}$$

6 $$\begin{aligned} P(\mathbf {X}^{\mathsf{i}}|U{=}b,\varPhi ,\mathbf{p})&= H^{\mathsf{out}}(\varPhi (\mathbf {X}^{\mathsf{o}}))/\eta _b, \end{aligned}$$

with $\eta _f= \sum _{j=1}^{N_\varPhi } \delta ^{\mathsf{on}}(\varPhi (\mathbf {X}^{\mathsf{o}}_j))$, and $\eta _b= \sum _{j=1}^{N_\varPhi } H^{\mathsf{out}}(\varPhi (\mathbf {X}^{\mathsf{o}}_j))$. The functions themselves, plotted in Fig. 5, are

7 $$\begin{aligned} \delta ^{\mathsf{on}}(\varPhi )&= \mathrm{sech}^2(\varPhi /2\sigma ) \end{aligned}$$

8 $$\begin{aligned} H^{\mathsf{out}}(\varPhi )&= {\left\{ \begin{array}{ll} 1-\delta ^{\mathsf{on}}(\varPhi ) \qquad &{}\text {if}\;\;\varPhi \ge {0} \\ 0 &{}\text {if}\;\;\varPhi <0. \end{array}\right. } \end{aligned}$$

The constant parameter $\sigma$ determines the width of the basin of attraction—a larger $\sigma$ gives a wider basin of convergence to the energy function, while a smaller $\sigma$ leads to faster convergence. In our experiments we use $\sigma = 2$.

The prior probabilities of observing foreground and background models $P(U=f)$ and $P(U=b)$ in Eq. (3) are assumed uniform:

9 $$\begin{aligned} P(U\!=\!f) \!=\! {\eta _f}/{\eta },\quad P(U\!=\!b) \!=\! {\eta _b}/{\eta },\quad \eta \!=\! \eta _f+ \eta _b~. \end{aligned}$$

Substituting Eqs. (5)–(9) into Eq. (3), the joint distribution for an individual pixel becomes

10 $$\begin{aligned}&P(\mathbf {X}^{\mathsf{i}},c,\varPhi ,\mathbf{p}) \nonumber \\&\quad =P(\varPhi ) P(\mathbf{p}) \left( P^{f}\delta ^{\mathsf{on}}(\varPhi (\mathbf {X}^{\mathsf{o}})) + P^{b}H^{\mathsf{out}}(\varPhi (\mathbf {X}^{\mathsf{o}}))\right) ~, \end{aligned}$$

where $$P^{f}{=}P(c|U{=}f) $$ and $$P^{b}{=}P(c|U{=}b)$$ are developed in Sect. 3.4 below.

### Pose Optimisation

Tracking involves determining the MAP estimate of the poses given their observed RGB-D images and the object shape $\varPhi$. We consider the pose at each time step *t* to be independent, and seek

11 $$\begin{aligned} {\text {argmax}}_{\mathbf{p}_t} P(\mathbf{p}_t | \varPhi , \varOmega _t) = {\text {argmax}}_{\mathbf{p}_t} \frac{P(\mathbf{p}_t, \varPhi , \varOmega _t)}{P(\varPhi , \varOmega _t)}~. \end{aligned}$$

Were the pose optimisation guaranteed to find the “correct” pose no matter what the starting state, this notion of independence would be exact. In practice it is an approximation. Assuming that tracking is healthy, to increase the chance of *maintaining* a correct pose we start the current optimization at the pose delivered at the previous time step, and accept that if tracking is failing this introduces bias. We note that the starting pose is not a prior, and we do not maintain a motion model.

The denominator in Eq. (11) is independent of $\mathbf{p}$ and can be ignored. (We drop the index *t* to avoid clutter). Because the image $\varOmega$ is sampled as a bag of pixels, we exploit pixel-wise independence and write the numerator as

12 $$\begin{aligned} P&(\mathbf{p}, \varPhi , \varOmega ) = \prod _{j=1}^{N_\varOmega } P(\mathbf {X}^{\mathsf{i}}_j,c_j,\varPhi ,\mathbf{p}) ~. \end{aligned}$$

Substituting $P(\mathbf {X}^{\mathsf{i}}_j,c_j,\varPhi ,\mathbf{p})$ from Eq. (10), and noting that $P(\varPhi )$ is independent of $\mathbf{p}$, and $P(\mathbf{p})$ will be uniform in the absence of prior information about likely poses,

13 $$\begin{aligned}&P(\mathbf{p}|\varPhi ,\varOmega ) \nonumber \\&\quad \sim \prod _{j=1}^{N_\varOmega } \left\{ {P^{f}_j\delta ^{\mathsf{on}}(\varPhi (\mathbf {X}^{\mathsf{o}}_j)) + P^{b}_jH^{\mathsf{out}}(\varPhi (\mathbf {X}^{\mathsf{o}}_j))} \right\} ~. \end{aligned}$$

The negative logarithm of Eq. (13) provides the cost

14 $$\begin{aligned} {\mathscr {E}}= -\sum _{j=1}^{N_\varOmega } \log \left\{ {P^{f}_j\delta ^{\mathsf{on}}(\varPhi (\mathbf {X}^{\mathsf{o}}_j)) + P^{b}_jH^{\mathsf{out}}(\varPhi (\mathbf {X}^{\mathsf{o}}_j))} \right\} \end{aligned}$$

to be minimised using Levenberg–Marquardt. In the minimisation, pose $\mathbf{p}$ is always set in a local coordinate frame, and the cost is therefore parametrised in the *change* in pose, ${\mathbf {p}^*}$. The derivatives required are

15 $$\begin{aligned} \frac{\partial {{\mathscr {E}}}}{\partial {\mathbf {p}^*}} = \sum _{j=1}^{N_\varOmega } \left\{ \left[ \frac{P^{f}_j\frac{\partial {\delta ^{\mathsf{on}}}}{\partial {\varPhi }}+P^{b}_j\frac{\partial {H^{\mathsf{out}}}}{\partial {\varPhi }}}{P(\mathbf {X}^{\mathsf{i}}_j,c_j|\varPhi ,\mathbf{p})} \frac{\partial {\varPhi }}{\partial {\mathbf {X}^{\mathsf{o}}_j}} \right] \frac{\partial {\mathbf {X}^{\mathsf{o}}_j}}{\partial {{\mathbf {p}^*}}}\right\} \end{aligned}$$

where $\mathbf {X}^{\mathsf{o}}$ is treated as a 3-vector. The derivatives involving $\delta ^{\mathsf{on}}$ and $H^{\mathsf{out}}$ are

16 $$\begin{aligned} \frac{\partial {\delta ^{\mathsf{on}}}}{\partial {\varPhi }} = -\frac{1}{\sigma }\mathrm{tanh}(\varPhi /2\sigma ) \mathrm{sech}^2(\varPhi /2\sigma ) \end{aligned}$$

and

17 $$\begin{aligned} \frac{\partial {H^{\mathsf{out}}}}{\partial {\varPhi }} = {\left\{ \begin{array}{ll} -\frac{\partial {\delta ^{\mathsf{on}}}}{\partial {\varPhi }} \qquad &{}\text {if}\;\;\varPhi \ge {0} \\ 0 &{}\text {if}\;\;\varPhi <0. \end{array}\right. } \end{aligned}$$

The derivatives $(\partial {\varPhi }/\partial {\mathbf {X}^{\mathsf{o}}})$ of the SDF are computed using finite central differences. We use modified Rodrigues parameters for the pose $\mathbf{p}$ (c.f. Shuster (1993)). Using the local frame, the derivatives of $\mathbf {X}^{\mathsf{o}}$ with respect to the pose update ${\mathbf {p}^*}=\left[ t^*_x,t^*_y,t^*_z,r^*_1,r^*_2,r^*_3\right] ^\top$ are always evaluated at identity so that

18 $$\begin{aligned} \frac{\partial {\mathbf {X}^{\mathsf{o}}}}{\partial {\,t_x^*}}&=\left[ \begin{array}{c}1\\ 0\\ 0\end{array}\right]&\frac{\partial {\mathbf {X}^{\mathsf{o}}}}{\partial {\,t_y^*}}&=\left[ \begin{array}{c}0\\ 1\\ 0\end{array}\right]&\frac{\partial {\mathbf {X}^{\mathsf{o}}}}{\partial {\,t_z^*}}&=\left[ \begin{array}{c}0\\ 0\\ 1\end{array}\right] \nonumber \\ \frac{\partial {\mathbf {X}^{\mathsf{o}}}}{\partial {\,r_1^*}}&=\left[ \begin{array}{c}0\\ -4Z^\mathsf{o}\\ 4Y^\mathsf{o}\end{array}\right]&\frac{\partial {\mathbf {X}^{\mathsf{o}}}}{\partial {\,r_2^*}}&=\left[ \begin{array}{c}4Z^\mathsf{o}\\ 0\\ -4X^\mathsf{o}\end{array}\right]&\frac{\partial {\mathbf {X}^{\mathsf{o}}}}{\partial {\,r_3^*}}&=\left[ \begin{array}{c}-4Y^\mathsf{o}\\ 4X^\mathsf{o}\\ 0\end{array}\right] . \end{aligned}$$

The pose change is found from the Levenberg–Marquardt update as

19 $$\begin{aligned} {\mathbf {p}^*}= \left\{ -\left[ \mathtt{J}^\top \mathtt{J} + \lambda {\mathrm {diag}}{\left[ \mathtt{J}^\top \mathtt{J}\right] }\right] ^{-1}\frac{\partial {{\mathscr {E}}}}{\partial {{\mathbf {p}^*}}}\right\} ~, \end{aligned}$$

where $\mathtt{J}$ is the Jacobian matrix of the cost function, and $\lambda$ is the non-negative damping factor adjusted at each iteration. Interpreting the solution vector ${\mathbf {p}^*}$ as an element in $\mathbb {SE}(3)$, and re-expressing as a $4\times 4$ matrix, we apply the incremental transformation at iteration $n+1$ onto the estimated transformation at the previous iteration *n* as $\mathtt{T}^{n+1}\leftarrow \mathtt{T}({\mathbf {p}^*})\mathtt{T}^{n}$. The estimated object pose $\texttt {T}^{\mathsf{oc}}$ results from composing the final incremental transformation $\mathtt{T}^{N}$ onto the previous pose as $\texttt {T}^{\mathsf{oc}}_{t+1}\leftarrow \mathtt{T}^{N}\texttt {T}^{\mathsf{oc}}_{t}$.

**Fig. 6:**
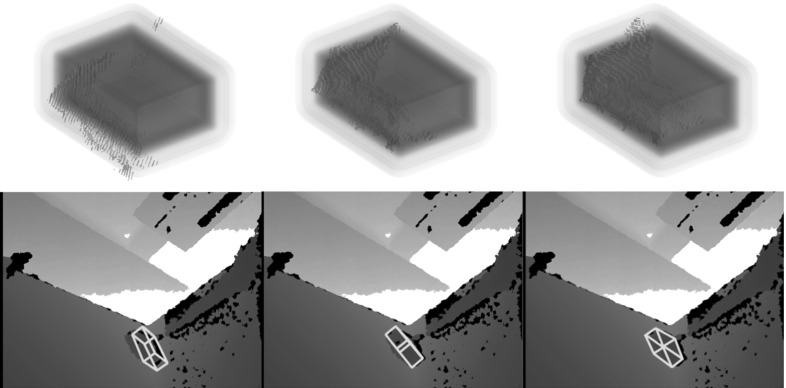
Typical process of convergence for one frame. The *top row* shows the back-projected points and the SDF in the object coordinates. The *bottom row* visualises the object outline on depth image with corresponding poses

**Fig. 7:**
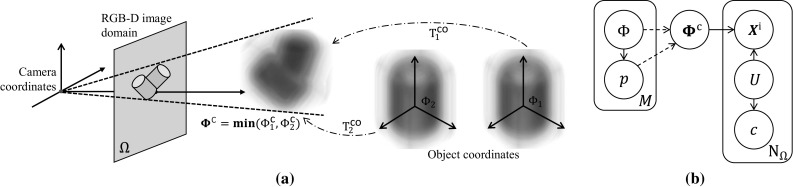
**a** Illustration of the fusion of multiple object SDFs in the shape union in the camera frame. SDFs are first transformed into camera coordinates then fused together by a minimum function. The observed RGB-D image domain is generated by projecting the fused SDF. **b** The extended graphical model

Figure 6 illustrates outputs from the tracking process during minimization. At each iteration the gradients of the cost function guide the back-projected points with $P^{f}>P^{b}$ towards the zero-level of the SDF and also force points with $P^{f}<P^{b}$ to move outside the object. At convergence, the points with $P^{f}>P^{b}$ will lie on the surface of the object.

The initial pose for the optimisation is specified manually or, in the case of live tracking, by placing the object in a prespecified position. An automatic technique, for example one based on regressing pose, could readily be incorporated to bootstrap the tracker.

### Online Learning of the Appearance Model

The foreground/background appearance model *P*(*c*|*U*) is important for the robustness of the tracking, and we adapt the appearance model online after tracking is completed on each frame. We use the pixels that have $|\varPhi (\mathbf {X}^{\mathsf{o}})|\le 3$ (that is, points that best fit the surface of the object) to compute the foreground appearance model and the pixels in the immediate surrounding region of the objects to compute the background model. The online update of the appearance model is achieved using a linear opinion pool

20 $$\begin{aligned} P_t(c|U=u) = (1-\rho ^u)P_{t-1}(c|U) + \rho ^u P_t(c|U) \end{aligned}$$

where $\rho ^u$ with $u \in \{f, b\}$ are the learning rates, set to $\rho ^{f} = 0.05$ and $\rho ^{b} = 0.3$. The background appearance model has a higher learning rate because we assume that the object is moving in an uncontrolled environment, where the change of appearance of the background is much faster than that of the foreground.

## Generalisation for Multiple Object Tracking

One straightforward approach to tracking multiple objects would be to replicate several single object trackers. However, as argued in the introduction and as shown below, a more careful approach is warranted. In Sect. 4.2 we will find a probabilistic way of resolving ambiguities in case of identical appearance models. Then in Sect. 4.3 we show how physical constraints such as collision avoidance can be incorporated in the formulation. First though we extend our notation and graphical model.

### Multi-Object Generative Model

The scene geometry and additional notation for simultaneous tracking of *M* objects is illustrated in Fig. 7(a), and the graphical generative model for the RGB-D image is shown in Fig. 7 (b). When tracking multiple objects in the scene, $\varOmega$ is conditionally dependent on the set of 3D object shapes $\{\varPhi _1\ldots \varPhi _M\}$ and their corresponding poses $\{\mathbf{p}_1\ldots \mathbf{p}_M\}$.

Given the shapes and poses at any particular time, we transform the shapes into the camera frame and fuse them into a single ‘shape union’ ${\varvec{\varPhi }}^{\mathsf {c}}$. Then, for each pixel location, the depth is drawn from the foreground/background model *U* and the shape union ${\varvec{\varPhi }}^{\mathsf {c}}$, following the same structure as in Sect. 3. The colour is drawn from the appearance model *P*(*c*|*U*), as before. We stress that although each object has a separate shape model in the set, two or more might be identical both in shape and appearance. This is the case later in the experiment of Fig. 14. We also note that when the number of objects drops to $M{=}1$ the generative model deflates gracefully to the single object case.

From the graphical model, the joint probability is

21 $$\begin{aligned}&P(\varPhi _1\ldots \varPhi _M,\mathbf{p}_1\ldots \mathbf{p}_M,{\varvec{\varPhi }}^{\mathsf {c}},\mathbf {X}^{\mathsf{i}},U,c) \nonumber \\&\quad = P(\varPhi _1\ldots \varPhi _M)P({\varvec{\varPhi }}^{\mathsf {c}}|\varPhi _1\ldots \varPhi _M,\mathbf{p}_1\ldots \mathbf{p}_M)\nonumber \\&\qquad P(\mathbf {X}^{\mathsf{i}},U,c |{\varvec{\varPhi }}^{\mathsf {c}})P(\mathbf{p}_1\ldots \mathbf{p}_M|\varPhi _1\ldots \varPhi _M) \end{aligned}$$

where

22 $$\begin{aligned} P(\mathbf {X}^{\mathsf{i}},U,c |{\varvec{\varPhi }}^{\mathsf {c}}) = P(\mathbf {X}^{\mathsf{i}}|U,{\varvec{\varPhi }}^{\mathsf {c}})P(c|U)P(U)~. \end{aligned}$$

Because the shape union is completely determined given the sets of shapes and poses, $P({\varvec{\varPhi }}^{\mathsf {c}}|\varPhi _1\ldots \varPhi _M,\mathbf{p}_1\ldots \mathbf{p}_M)$ is unity. As in the single object case, the posterior distribution of the set of poses given all object shapes can be obtained by marginalising over the latent variable *U*

23 $$\begin{aligned}&P(\mathbf{p}_1\ldots \mathbf{p}_M|\mathbf {X}^{\mathsf{i}},c,\varPhi _1\ldots \varPhi _M)\sim \nonumber \\&\quad P(\mathbf {X}^{\mathsf{i}},c|{\varvec{\varPhi }}^{\mathsf {c}})P(\mathbf{p}_1\ldots \mathbf{p}_M|\varPhi _1\ldots \varPhi _M)~, \end{aligned}$$

where

24 $$\begin{aligned}&P(\mathbf {X}^{\mathsf{i}},c|{\varvec{\varPhi }}^{\mathsf {c}})\nonumber \\&\quad =\sum _{u\in \{f,b\}}P(\mathbf {X}^{\mathsf{i}}|U=u,{\varvec{\varPhi }}^{\mathsf {c}})P(c|U=u)P(U=u). \end{aligned}$$

The first term in Eq. (23), $P(\mathbf {X}^{\mathsf{i}},c|{\varvec{\varPhi }}^{\mathsf {c}})$, describes how likely a pixel is to be generated by the current shape union, in terms of both the colour value and the 3D location, and is referred to as the data term. The second term, $P(\mathbf{p}_1\ldots \mathbf{p}_M|\varPhi _1\ldots \varPhi _M)$, puts a prior on the set of poses given the set of shapes and provides a physical constraint term.

### The Data Term

Echoing Sect. 3, the per-pixel likelihoods $P(\mathbf {X}^{\mathsf{i}}|U=u,{\varvec{\varPhi }}^{\mathsf {c}})$ are defined by smoothed delta and Heaviside functions

25 $$\begin{aligned} P(\mathbf {X}^{\mathsf{i}}|U=f, {\varvec{\varPhi }}^{\mathsf {c}})&= {\delta ^{\mathsf{on}}({\varvec{\varPhi }}^{\mathsf {c}}(\mathbf {X}^{\mathsf{c}}))}/{\eta _f^\mathsf{c}} \end{aligned}$$

26 $$\begin{aligned} P(\mathbf {X}^{\mathsf{i}}|U=b,{\varvec{\varPhi }}^{\mathsf {c}})&= {H^{\mathsf{out}}({\varvec{\varPhi }}^{\mathsf {c}}(\mathbf {X}^{\mathsf{c}}))}/{\eta _b^\mathsf{c}} \end{aligned}$$

where $\eta _f^\mathsf{c} = \sum _{j=1}^{N_\varOmega } \delta ^{\mathsf{on}}({\varvec{\varPhi }}^{\mathsf {c}}(\mathbf {X}^{\mathsf{c}}_j))$, $\eta _b^\mathsf{c} = \sum _{j=1}^{N_\varOmega } H^{\mathsf{out}}({\varvec{\varPhi }}^{\mathsf {c}}(\mathbf {X}^{\mathsf{c}}_j))$, and where $\mathbf {X}^{\mathsf{c}}$ is the back-projection $\mathbf {X}^{\mathsf{i}}$ into the camera frame (note, not the object frame). The per-pixel labellings again follow uniform distributions

27 $$\begin{aligned} P(U\!=\!f) \!=\! \frac{\eta _f^\mathsf{c}}{\eta ^\mathsf{c}}, \quad P(U= & {} b) \!=\! \frac{\eta _b^\mathsf{c}}{\eta ^\mathsf{c}}, \quad \eta ^\mathsf{c} = \eta _f^\mathsf{c} \!+\! \eta _b^\mathsf{c}. \end{aligned}$$

Substituting Eqs. (25–27) into Eq. (24) we obtain the likelihood of the shape union for a single pixel

28 $$\begin{aligned} P(\mathbf {X}^{\mathsf{i}},c|{\varvec{\varPhi }}^{\mathsf {c}}) = P^{f}\delta ^{\mathsf{on}}({\varvec{\varPhi }}^{\mathsf {c}}(\mathbf {X}^{\mathsf{c}})) + P^{b}H^{\mathsf{out}}({\varvec{\varPhi }}^{\mathsf {c}}(\mathbf {X}^{\mathsf{c}})), \end{aligned}$$

where $P^{f}$ and $P^{b}$ are the appearance models of Sect. 3.

To form the shape union ${\varvec{\varPhi }}^{\mathsf {c}}$ we transform each object shape $\varPhi _m$ into camera coordinates as $\varPhi ^{\mathsf {c}}_m$ using $\texttt {T}^{\mathsf{co}}(\mathbf{p}_m)$, and fuse them into a single SDF with the minimum function approximated by an analytical relaxation

29 $$\begin{aligned} {\varvec{\varPhi }}^{\mathsf {c}}= \min {\left( \varPhi ^{\mathsf {c}}_1,\ldots ,\varPhi ^{\mathsf {c}}_M\right) } \;\approx \; -\frac{1}{\alpha }\log {\sum _{m=1}^M{\exp \{-\alpha \varPhi ^{\mathsf {c}}_m}}\} \end{aligned}$$

in which $\alpha$ controls the smoothness of the approximation. Larger $\alpha$ gives a better approximation of the minimum function, but we find empirically that choosing a smaller $\alpha$ gives a wider basin of convergence for the tracker. We use $\alpha {=}2$ in this work. The per-voxel values of $\varPhi ^{\mathsf {c}}_m$ are calculated using

30 $$\begin{aligned} \varPhi ^{\mathsf {c}}_m(\mathbf {X}^{\mathsf{c}}) =\varPhi _m(\mathbf {X}^{\mathsf{o}}_m) \end{aligned}$$

where $\mathbf {X}^{\mathsf{o}}_m = \texttt {T}^{\mathsf{oc}}(\mathbf{p}_m)\mathbf {X}^{\mathsf{c}}$ is the transformation of $\mathbf {X}^{\mathsf{c}}$ into the *m*-th object’s frame. The likelihood for a pixel then becomes

31 $$\begin{aligned}&P(\mathbf {X}^{\mathsf{i}},c|{\varvec{\varPhi }}^{\mathsf {c}}) \nonumber \\&\quad =P^{f}\delta ^{\mathsf{on}}\left( -\frac{1}{\alpha }\log {\sum _{m=1}^M {\exp \{-\alpha \varPhi _m(\mathbf {X}^{\mathsf{o}}_m)}}\}\right) \nonumber \\&\qquad \quad +P^{b}H^{\mathsf{out}}\left( -\frac{1}{\alpha }\log {\sum _{m=1}^M{\exp \{- \alpha \varPhi _m(\mathbf {X}^{\mathsf{o}}_m)}}\}\right) ~. \end{aligned}$$

Assuming pixel-wise independence, the negative log likelihood across the RGB-D image provides a data term

32 $$\begin{aligned} \mathscr {E}_{{\text {data}}}= -\log P(\varOmega |{\varvec{\varPhi }}^{\mathsf {c}}) = -\sum _{j=1}^{N_\varOmega }\log P(\mathbf {X}^{\mathsf{i}}_j,c|{\varvec{\varPhi }}^{\mathsf {c}}) \end{aligned}$$

in the overall energy function.

We will require the derivatives of this term w.r.t. the change of the set of pose parameters ${\varvec{\varTheta }}^*{=}\{\mathbf{p}_1^*\ldots \mathbf{p}_M^*\}$. Dropping the pixel index *j*, we write

33 $$\begin{aligned} \frac{\partial {\mathscr {E}_{{\text {data}}}}}{\partial {{\varvec{\varTheta }}^*}}&= -\sum _{\mathbf {X}^{\mathsf{i}}\in \varOmega }\left\{ \frac{P^{f}\frac{\partial {\delta ^{\mathsf{on}}}}{\partial {{\varvec{\varPhi }}^{\mathsf {c}}}} +P^{b}\frac{\partial {H^{\mathsf{out}}}}{\partial {{\varvec{\varPhi }}^{\mathsf {c}}}}}{P(\mathbf {X}^{\mathsf{i}},c|{\varvec{\varPhi }}^{\mathsf {c}})} \frac{\partial {{\varvec{\varPhi }}^{\mathsf {c}}(\mathbf {X}^{\mathsf{c}})}}{\partial {{\varvec{\varTheta }}^*}} \right\} \end{aligned}$$

where

34 $$\begin{aligned} \frac{\partial {{\varvec{\varPhi }}^{\mathsf {c}}(\mathbf {X}^{\mathsf{c}})}}{\partial {{\varvec{\varTheta }}^*}}&= -\frac{1}{\alpha } \sum _{m=1}^{M} w_m \frac{\partial {\varPhi _m}}{\partial {\mathbf {X}^{\mathsf{o}}_m}} \frac{\partial {\mathbf {X}^{\mathsf{o}}_m}}{\partial {{\varvec{\varTheta }}^*}}~, \end{aligned}$$

35 $$\begin{aligned} w_m&=\frac{\exp \{-\alpha \varPhi _m(\mathbf {X}^{\mathsf{o}}_m)\}}{\sum _{k=1}^{M}\exp \{-\alpha \varPhi _k(\mathbf {X}^{\mathsf{o}}_k)\}} , \end{aligned}$$

and

36 $$\begin{aligned} \frac{\partial {\mathbf {X}^{\mathsf{o}}_m}}{\partial {{\varvec{\varTheta }}^*}} = \left[ \frac{\partial {\mathbf {X}^{\mathsf{o}}_m}}{\partial {\mathbf{p}_1^*}}\ldots \frac{\partial {\mathbf {X}^{\mathsf{o}}_m}}{\partial {\mathbf{p}_M^*}}\right] ~. \end{aligned}$$

The remaining pose and SDF derivatives ($\partial {\mathbf {X}^{\mathsf{o}}_m}/\partial {\mathbf{p}_k^*}$ and $\partial {\varPhi _m}/\partial {\mathbf {X}^{\mathsf{o}}_m}$) are as in Sect. 3.

Note that instead of assigning a pixel $\mathbf {X}^{\mathsf{i}}$ in the RGB-D image domain deterministically to one object, we back-project $\mathbf {X}^{\mathsf{i}}$ (i.e. $\mathbf {X}^{\mathsf{c}}$ in camera coordinates) into all objects’ frames with the current set of poses. The weights $w_m$ are then computed according to Eq. (35), giving a smoothly varying pixel to object association weight. This can also be interpreted as the probability that a pixel is projected from the *m*-th object. If the back-projection $\mathbf {X}^{\mathsf{o}}_m$ of $\mathbf {X}^{\mathsf{c}}$ is close to the *m*-th object’s surface ($\varPhi (\mathbf {X}^{\mathsf{o}}_m) \approx 0$) and other back-projections $\mathbf {X}^{\mathsf{o}}_k$ are further away from the surfaces ($\varPhi (\mathbf {X}^{\mathsf{o}}_k) \gg 0$), then we will find $w_m \rightarrow {1}$ and the other $w_k \rightarrow 0$.

### Physical Constraint Term

Consider $P(\mathbf{p}_1\ldots \mathbf{p}_M|\varPhi _1\ldots \varPhi _M)$ in Eq. (24). We decompose the joint probability of all object poses given all 3D object shapes into a product of per-pose probabilities:

37 $$\begin{aligned}&P(\mathbf{p}_1\ldots \mathbf{p}_M|\varPhi _1\ldots \varPhi _M)\nonumber \\&\quad =P(\mathbf{p}_1|\varPhi _1\ldots \varPhi _M)\prod _{m=2}^{M}P(\mathbf{p}_m| \{\mathbf{p}\}_{-m}, \varPhi _1\ldots \varPhi _M) \end{aligned}$$

where $\{\mathbf{p}\}_{-m}= \{\mathbf{p}_1 \ldots \mathbf{p}_M\} \setminus \{\mathbf{p}_m\}$ is the set of poses excluding $\mathbf{p}_m$. We do not place any pose priors on any single objects, so we can ignore the factor $P(\mathbf{p}_1|\varPhi _1\ldots \varPhi _M)$. The remaining factors can be used to enforce pose-related constraints.

Here we use them to avoid object collisions by discouraging objects from penetrating each other. The probability $P(\mathbf{p}_m|\{\mathbf{p}\}_{-m}, \varPhi _1\ldots \varPhi _M)$ is defined such that a surface point on one object should not move inside any other object. For each object *m* we uniformly and sparsely sample a set of *K* “collision points” $\mathscr {C}_m =\{\mathbf {C}^{\mathsf{o}}_{m,1}\dots \mathbf {C}^{\mathsf{o}}_{m,K}\}$ from its surface in object coordinates. *K* needs to be high enough to account for the complexity of the tracked shape, and not undersample parts of the model. We found throughout our experiments that $K=1000$ insures sufficient coverage of the object to produce an effective collision constraint.

At each timestep the collision points are transformed into the camera frame as $\{\mathbf {C}^{\mathsf{c}}_{m,1}\ldots \mathbf {C}^{\mathsf{c}}_{m,K}\}$ using the current pose $\mathbf{p}_m$. Denoting the partial union of SDFs $\{\varPhi ^{\mathsf {c}}_{1}\ldots \varPhi ^{\mathsf {c}}_{M}\} \setminus \{ \varPhi ^{\mathsf {c}}_{m} \}$ by ${\varvec{\varPhi }}^{\mathsf {c}}_{-m}$ we write

38 $$\begin{aligned} P(\mathbf{p}_m | \{\mathbf{p}\}_{-m}, \varPhi _1\ldots \varPhi _M) \sim \frac{1}{K}\sum _{k=1}^{K} H^{\mathsf{out}}\left( {\varvec{\varPhi }}^{\mathsf {c}}_{-m}(\mathbf {C}^{\mathsf{c}}_{m,k})\right) \end{aligned}$$

where $H^{\mathsf{out}}$ is the offset smoothed Heaviside function already defined. If all the collision points on object *m* lie outside the shape union of objects excluding *m* this quantity asymptotically approaches 1. If progressively more of the collision points lie inside the partial shape union, the quantity asymptotically approaches 0.

The negative log-likelihood of Eq. (38) gives us the second part of the overall cost

39 $$\begin{aligned} \mathscr {E}_{{\text {coll}}}= -\sum _{m=1}^{M}\log \left( \frac{1}{K}\sum _{k=1}^{K} {H^{\mathsf{out}}\big ({\varvec{\varPhi }}^{\mathsf {c}}_{m-}(\mathbf {C}^{\mathsf{c}}_{m,k})\big )}\right) . \end{aligned}$$

The derivatives of this energy are computed analogously to those used for the data term (Eqs. 33 and 34), but with ${\varvec{\varPhi }}^{\mathsf {c}}(\mathbf {X}^{\mathsf{c}})$ replaced by ${\varvec{\varPhi }}^{\mathsf {c}}_{m-}(\mathbf {C}^{\mathsf{c}}_{m,k})$.

### Optimisation

The overall cost is the sum of the data term and the collision constraint term

40 $$\begin{aligned} {\mathscr {E}}= \mathscr {E}_{{\text {data}}}+ \mathscr {E}_{{\text {coll}}}. \end{aligned}$$

To optimise the set of poses $\{\mathbf{p}_1\ldots \mathbf{p}_M\}$, we use the same Levenberg-Marquardt iterations and local frame pose updates as given in Sect. 3.

**Fig. 8:**
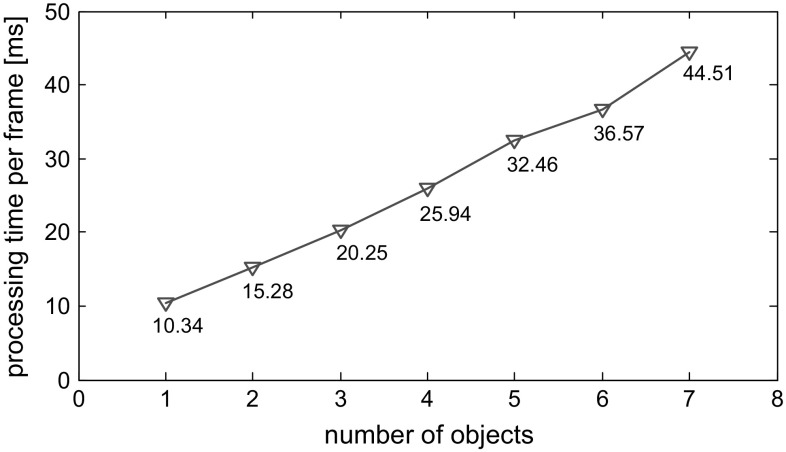
The processing time per frame in milliseconds of the multi-object tracker implemented on the CPU rises linearly with the the number of objects tracked

**Fig. 9:**
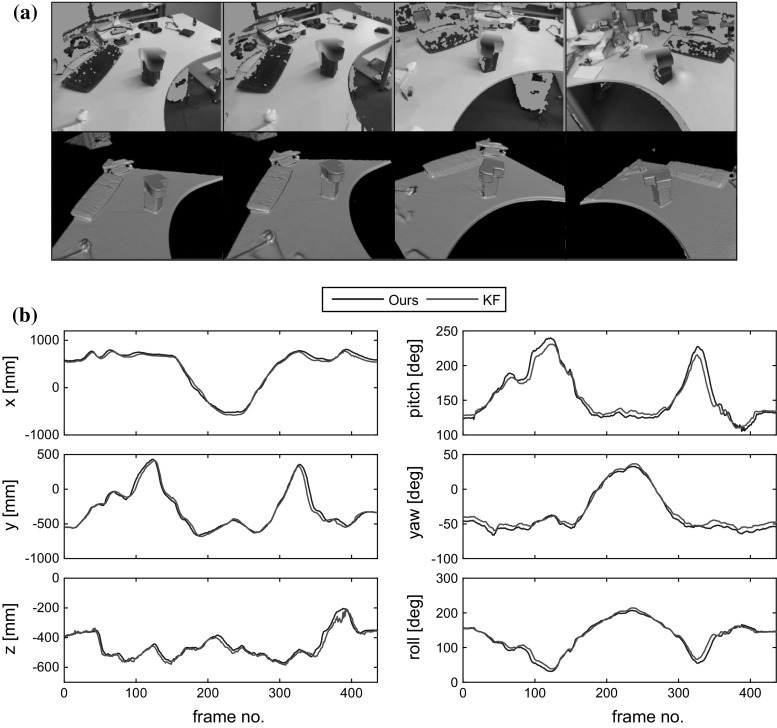
A quantitative comparison of camera pose output obtained using the present method on a single object and from using KinectFusion on the entire scene. **a** Frames from the two approaches. *Top row* the tracked object using our method. *Bottom row* camera track from KinectFusion. **b** The 6 degrees of freedom in pose compared. Translation is measured in mm and rotation is measured in degrees.

## Implementation

We have coded separate CPU and GPU versions of our generalised multi-object tracker. Figure 8 shows the processing time per frame for the CPU implementation executing on an Intel Core i7 3.5 GHz processor with OpenMP support as the number of objects tracked is increased. As expected, the time rises linearly with the number of objects. With two objects the CPU version runs at around 60 Hz, but above five objects the process is at risk of falling below frame rate. The accelerated version, running on an Nvidia GTX Titan Black GPU and same CPU, typically yields a 30% speed-up in the experiments reported below. The rate is not greatly increased because the GPU only applies full leverage to image pixels that backproject into the 3D voxelised volumes around objects. In the experiments here, the tracked objects typically occupy a very small fraction (i.e. just a few %) of the RGB-D image, involving only a few thousands of pixels, insufficient to exploit massive parallelism.

## Experiments

We have performed a variety of experimental evaluations, both qualitative and quantitative. Qualitative examples of our algorithm tracking different types of objects in real-time and under significant occlusion and missing data can be found in the video at https://youtu.be/BSkUee3UdJY. (NB: to be replaced by an official archival site).

**Fig. 10:**
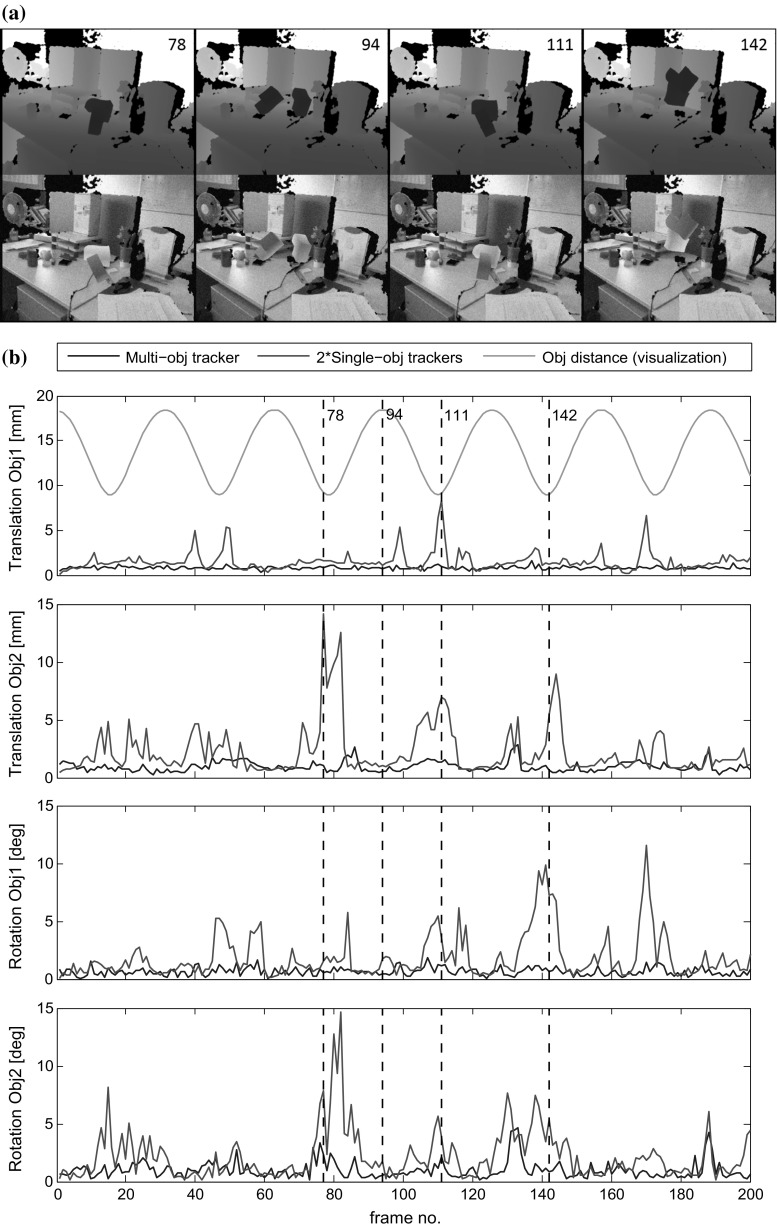
A comparison of pose estimation error between our generalised multi-object tracker and two instances of our single object method. **a** Four examples of the synthetic RGB-D frames with the frame number corresponding to the marks on the pose graphs in **b**. **b** As the objects are periodically brought closer, so the pose error (*red*) of the two independent trackers increases (Color figure online)

### Quantitative Experiments

We ran three sets of experiments to benchmark the tracking accuracy of our algorithms. First we compare the camera trajectory obtained by our algorithm tracking a single stationary object against that obtained by the KinectFusion algorithm of Newcombe et al. (2011) tracking the entire world map. Several frames from the sequence used are shown in Fig. 9a and the degrees of freedom in translation and rotation are compared in Fig. 9b. Despite using only the depth pixels corresponding to the object (an area of the depth image considerably smaller than that employed by KinectFusion) our algorithm obtains comparable accuracy. It should be noted that this is not a measure of ground truth accuracy: the trajectory obtained by the KinectFusion is itself just an estimate.

**Fig. 11:**
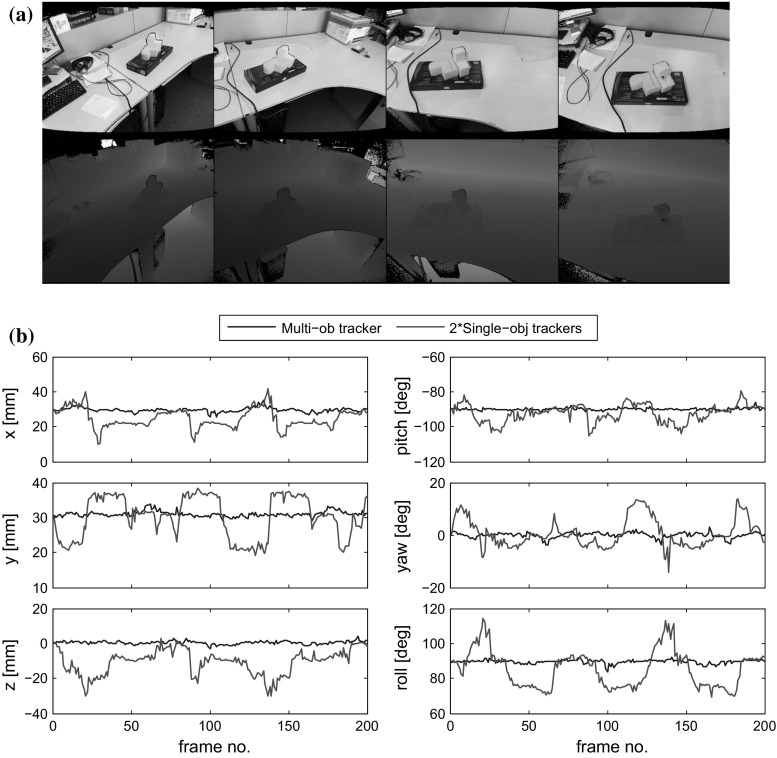
Comparison of the difference in relative pose estimation between our multi-object tracker and two instances of our single-object tracker using real data. **a** Sample frames **b** Pose recovery compared: the multiobject tracker (*blue*) is stable (Color figure online)

In our second experiment, we follow a standard benchmarking strategy from the markerless tracking literature and evaluate our tracking results on synthetic data to provide ground truth. We move two objects of known shape in front of a virtual camera and generate RGB-D frames. The objects periodically move further apart then closer to each other. Realistic levels of Gaussian noise are added to both the rendered colour and the depth images. Four sample frames from the test sequence are shown in Fig. 10a. Using this sequence we compare the tracking accuracy of our generalised multi-object tracker with two instances of our single object tracker. To evaluate translation accuracy we use the Euclidean distance between the estimated and ground truth poses. To measure rotation accuracy, we rotate the unit vectors to the three axis directions $\mathbf {e}_x$,$\mathbf {e}_y$,$\mathbf {e}_z$ using the ground truth $\mathtt {R}_g$ and we estimate the rotation matrix $\mathtt {R}_e$. The error value is averaged over the three including angles of the resulting vectors:

41 $$\begin{aligned} r_{ err } = \frac{1}{3}\sum _{i\in {x,y,z}}\cos ^{-1}\left( (\mathtt {R}_e\mathbf {e}_i)^\top \mathtt {R}_g\mathbf {e}_i)\right) \end{aligned}$$

In the graphical results of Fig. 10b the green line shows the relative distance between the two objects. Note that this value has been scaled and offset for visualisation. It can be seen that when the two objects with similar appearance model are neither overlapping nor close (e.g. frame 94), both two single object trackers and multi-object tracker provide accurate results. However, once the two objects move close together, the two separate single object trackers produce large errors. The single object tracker fails to model the pixel membership, leading to an incorrect pixel association when the two objects are close together. Our soft pixel membership solves this problem.

The third quantitative experiment (Fig. 11) makes a similar comparison, but with real imagery. As before, it is difficult to obtain the absolute ground truth pose of the objects, and instead we measure the consistency of the relative pose between two static objects by moving the camera around while looking towards the two objects. Example frames are shown in Fig. 11a. If the two recovered poses are accurate we would expect consistent relative translation and rotation through the whole sequence. As shown in Fig. 11b, our multi-object tracker is able to recover much more consistent relative translation and rotation than two independent instances of our single object tracker.

**Fig. 12:**
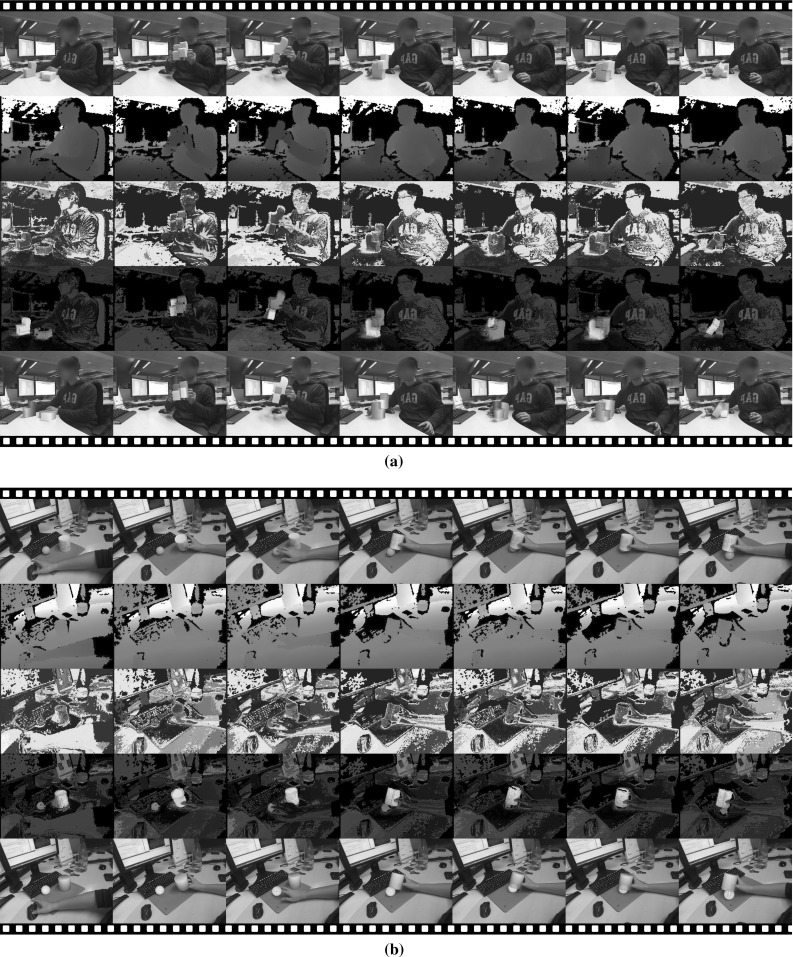
Film strips showing our algorithm tracking accurately known object models: **a** two pieces of formed sponge, and **b** a ball and a cup. In each, *Rows 1&2* show the *colour* and the depth image inputs. *Row 3* is per-pixel foreground probability $P^{f}$. *Row 4* is the per-pixel membership weight $w_i$, *magenta and cyan* olour correspond to the two objects, the *blue coloured* pixels are with ambiguous membership. *Row 5* shows the tracking result (Color figure online)

**Fig. 13:**
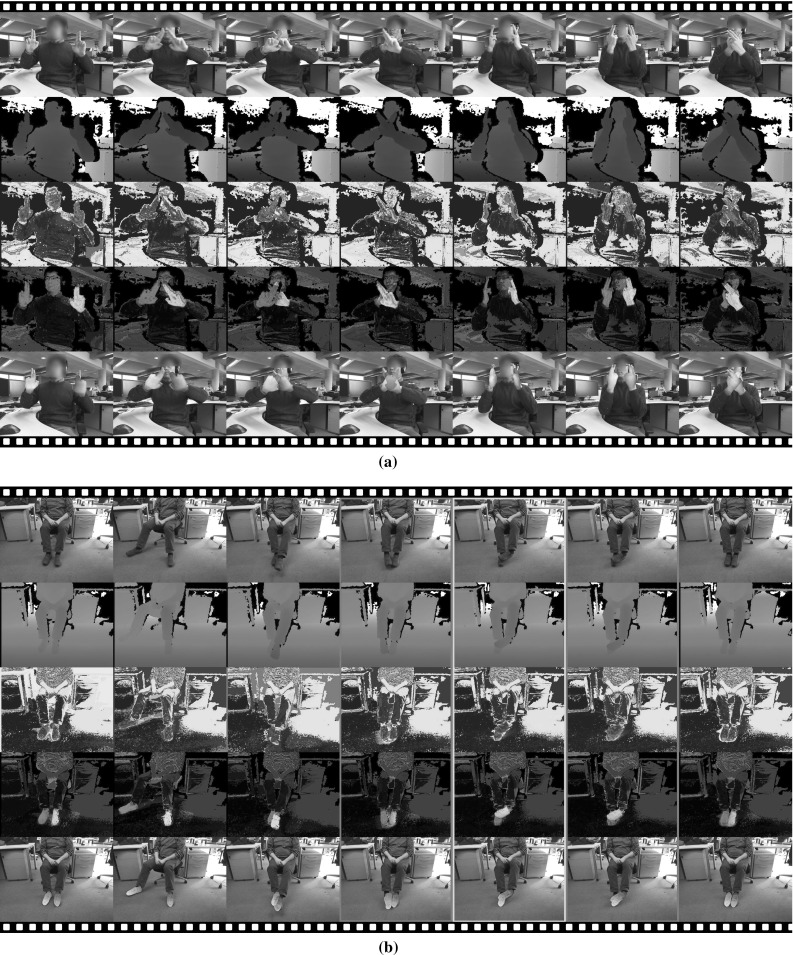
Film strip showing our algorithm tracking two cases where the models are inaccurate: **a** two hands and **b** two feet. The rows are as in Fig. 12

**Fig. 14:**
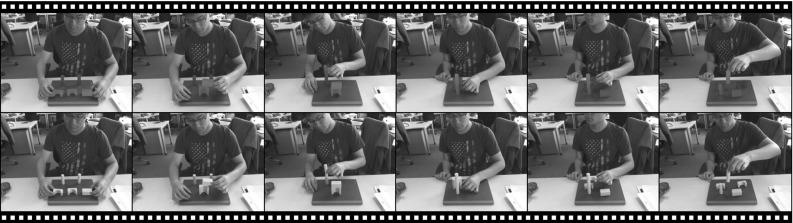
A film strip showing a very challenging sequence where 5 pieces of toy bricks with identical *colour* are tracked. The top sequence shows the tracking result rendered on the *colour* image and the sequence below shows the original *colour* images. Our multi-object tracker manages to track through the whole sequence without tracking failure

### Qualitative Experiments

We use five challenging real sequences to illustrate the robust performance of our multi-object tracker.

In Fig. 12 we use *accurate*, hand crafted models for tracking. Figure 12a shows the tracking of two pieces of sponge with identical shape and appearance models. Rows 1 and 2 of the figure show the colour and the depth image inputs, and Row 3 shows the per-pixel foreground probability $P^{f}$. Row 4 shows the per-pixel membership weight $w_m$. The two objects, one with $w_1\gg 0.5$, $w_2\ll 0.5$ and the other with $w_2\gg 0.5$, $w_1\ll 0.5$, are highlighted in magenta and cyan respectively. The blue highlighted pixels have ambiguous membership ($w_1\approx w_2\approx 0.5$). The darkened pixels are background, as obvious from Row 3. The final tracking result is shown as Row 5.

The tracker is able to track through heavy occlusions and handle challenging motions. This is a result of the region based nature of our approach, which makes it robust to missing or occluded parts of the tracked target, as long as these do not introduce extra ambiguity in the shape to pose mapping.

In Fig. 12b we simultaneously track a white cup and a white ball to demonstrate the effectiveness of the physical collision constraint. Even though there is no depth observation from the ball owing to significant occlusion from the cup, our algorithm can still estimate the location of the ball. This happens because (i) the physical constraint prevents the ball from intersecting with the cup and (ii) the table is a different colour from the ball, which prevents the ball from overlapping with the table.

As a contrast, Fig. 13 illustrates our tracker using previously reconstructed and hence somewhat *inaccurate* 3D shapes. First in Fig. 13a we track two interacting hands (fixed hand articulation pose). Even though the hand models do not fit the observation perfectly—indeed they are models of hands from a different person obtained using the algorithm (Ren et al. 2013)—the tracker still recovers the poses of both by finding the local minimum that best explains the colour and depth observations.

In Fig. 13b we track two interacting feet with a pair of approximate shoe models. Throughout most of the sequence our tracker successfully recovers the two poses. However, we do also encounter two failure cases here. The first one is shown in column 4 of Fig. 13b, where the shoe is incorrectly rotated. This happens because the 3D model is somewhat rotationally ambiguous around its long axis. The second failure case can be seen in Column 6. Here, the ground pixels (i.e. the black shadow) have very high foreground probability, as can be clearly seen in Row 3. With most of one foot occluded, the tracker incorrectly tries to fit the model to the pixels with high foreground probability, leading to failure. We note that the tracker does automatically recover from both failure cases. As soon as the feet move out of the ambiguous position, the multi-object tracker uses the previous incorrect pose as initialization and converges to the correct pose at the current frame.

In Fig. 14 we show a challenging sequence where five toy bricks are tracked, illustrating that the proposed tracker is able to handle larger number of objects. All the objects in the toy set have the same colour and some also have identical shapes. The top sequence shows the tracking result and the bottom sequence shows the original colour input. In spite of the heavy self-occlusion and the occlusion introduced by hands, the multi-object tracker is able to track robustly. Importantly, there is no bleeding of one object into another when blocks are placed together then separated.

## Conclusions

In this paper we presented a novel framework for tracking single and multiple 3D objects from a sequence of RGB-D images. Our method is particularly well suited to tracking several objects with similar or identical appearance, which is a common case in many applications, such as tracking cars or pairs of hands or feet. Our method is grounded in a rigorous probabilistic framework, yielding weights that indicate the probability of individual image observations being generated by each of the tracked objects, thus implicitly solving the data association problem. Furthermore, in the multi-object case, the formulation leads to a natural imposition of a physical constraint term, allowing us to specify prior knowledge about the world. We have used this term to indicate that it is unlikely that several objects occupy the same locations in 3D space. In addition to collision avoidance, the formulation would allow for generic interaction forces between objects to be modelled.

We validate our claims with several experiments, showing both robustness and accuracy. For this evaluation we used an implementation that can easily track multiple objects in real-time without the use of any GPU acceleration.

Since the tracker is region-based and currently uses simple histograms as appearance models, it is particularly well suited to objects where the texture is uninformative. A possible direction of research is to transfer our tracking framework to different appearance models, such as texture-based models. In line with other model-based 3D trackers our approach currently also requires 3D models of the tracked objects to be known and given to the algorithm. While we do explicitly show good performance even with crude and inaccurate models, this might be considered another shortcoming to be resolved in future work. In particular, dynamic objects such as hands could be an interesting area to explore further, as tracking individual fingers might greatly benefit from a method that can handle near-identical appearance and imposes collision constraints.
